# Linking Parenting Styles and Practices to Anxiety and Physical Activity in Autistic Youth: A Mediation Model

**DOI:** 10.3390/children12111510

**Published:** 2025-11-07

**Authors:** Yosi Yaffe, Michal Ben-Eli, Orna Huri, Batel Hazan-Liran, Orr Levental

**Affiliations:** 1 Department of Special Education, Tel Hai Academic College, Kiryat Shmona 12208, Israelbatellir@telhai.ac.il (B.H.-L.); 2 Department of Physical Education, Tel Hai Academic College, Kiryat Shmona 12208, Israel

**Keywords:** parenting styles, ASD, physical activity, anxiety

## Abstract

**Background/Objectives:** Individuals with autism spectrum disorder (ASD) often experience high anxiety and low physical activity (PA). While the influence of parenting styles on these outcomes is well-documented in typically developing children, their role in autistic youth remains underexplored. The study examines how parenting style and parental encouragement of physical activity relate to anxiety and activity levels in ASD youth. **Methods:** The sample consisted of 76 parents of school-aged children diagnosed with ASD, including 54 parents of boys and 22 parents of girls (Aged 6–18; Mage = 10.75, SD = 3.67). The parents’ ages ranged from 23 to 65 years (*M* = 42.96, SD = 7.01). **Results:** Using a path model analysis, we found that authoritarian and permissive parenting were directly associated with elevated child anxiety. Authoritative and permissive parenting were inversely associated with child anxiety indirectly via parental encouragement of PA. Furthermore, authoritative and permissive parenting were inversely associated with the child’s PA score via encouragement of PA. **Conclusions:** The study establishes links between parenting styles and anxiety and physical activity in ASD children and adolescents, while identifying a specific mechanism that partially explains these associations.

## 1. Introduction

Physical activity (PA) is widely acknowledged as crucial for maintaining physical and psychological health, contributing significantly to overall quality of life across diverse age groups. Regular engagement in PA reduces risks associated with chronic illnesses, enhances cardiovascular functions, promotes cognitive health, including memory and attention, and mitigates risks for depression and anxiety [1,2]. It also fosters social integration, emotional regulation, self-esteem, and resilience, particularly when conducted within social or team-based contexts [3,4]. Although most research examining the association between PA and emotional well-being has focused on typically developing populations, there is growing recognition of the potential benefits PA may also offer to individuals with neurodevelopmental disorders, including autism spectrum disorder (ASD). Regular participation in adapted PA has been shown to enhance social skills and reduce common social barriers experienced by ASD youth, indirectly contributing to decreased social isolation and social anxiety [5]. Adapted physical activity for individuals with ASD have been associated with improved emotional outcomes, including reduced anxiety levels, enhanced mood, and greater overall mental well-being [6,7].

ASD is characterized by persistent impairments in social communication and interactions, alongside restrictive, repetitive behaviors and sensory sensitivities [8]. Individuals with ASD frequently experience heightened psychological vulnerabilities, including elevated rates of anxiety, depression, and behavioral difficulties [9,10]. Anxiety is widely recognized as a prevalent and pervasive emotional burden among young individuals with ASD, with various anxiety disorders affecting approximately 40% of this population [11]. This elevated prevalence underscores the substantial emotional vulnerability associated with ASD and highlights anxiety as a core comorbid condition requiring focused clinical and research attention. These emotional and psychopathological challenges significantly impact their adaptive functioning and social–communicative abilities, further complicating personal adjustment and societal integration. Despite the evident benefits of PA, research consistently indicates lower participation rates among individuals with ASD compared to their neurotypical peers [12]. Factors contributing to reduced activity in this population include motor skill deficits, sensory processing difficulties, and inherent social challenges that create barriers to engaging comfortably in group or recreational activities [13,14].

Parenting practices and styles represent a fundamental ecological factor that profoundly shapes children’s emotional and psychological functioning, playing a critical role not only in the development of adaptive emotion-regulation capacities and in the modulation of anxiety symptoms across developmental stages, but also in facilitating and sustaining health-promoting behaviors such as regular engagement in PA. Thus, parenting plays an important role in shaping both mental well-being and physical health in children and adolescents. A substantial body of research on childhood and adolescent anxiety highlights the central etiological role of parenting styles and practices marked by low warmth and high control. Specifically, parenting characterized by strict discipline, excessive behavioral regulation, overprotectiveness, autonomy suppression, and intrusive psychological control has been consistently linked to elevated anxiety symptoms and anxiety disorders in youth [15,16,17,18]. These maladaptive parenting behaviors may function as direct contributors to the development of anxiety, as reactive responses to the child’s existing anxiety, or as manifestations of the parents’ own anxious dispositions. Moreover, parenting practices may influence children’s anxiety indirectly by encouraging or discouraging certain child behaviors that, in turn, increase vulnerability to anxiety-related outcomes [19,20]. Such is the case with offspring with disabilities, among which parenting practices and behaviors were also linked to increased anxiety symptoms. Indeed, children and adolescents with ASD and Down Syndrome (DS) raised in contexts of greater psychological control or inconsistent/permissive discipline tended to show higher and various anxiety symptoms [21,22,23]. Yet, despite strong theories linking parenting style to child anxiety, very little empirical work has tested this connection in autism. Only a few quantitative studies have included autistic samples, while cross-sectional, longitudinal, or multi-informant designs are almost absent. This scarcity means current clinical guidance still rests mainly on findings from typically developing youth, underscoring a major evidence gap for understanding and treating anxiety in ASD.

Especially in the context of atypical developmental conditions, understanding how parental styles and behaviors, particularly parental encouragement of PA, influence the PA engagement of young people with ASD, and subsequently their levels of anxiety, emerges as a significant area for further exploration. This line of inquiry holds potential implications not only for enhancing participation in PA but also for optimizing emotional health and overall quality of life within this unique population. Recent empirical information suggests that parenting styles and PA-related practices and influences can considerably foster motivation towards and promote actual participance in PA among children and adolescents [24,25]. Indeed, parental PA practices such as encouragement, support, and modeling were recognized in several meta-analytic works as factors associated with increased PA and various other health behaviors predominantly in typical developing young people [26,27]. However, more empirical information is required in the research literature regarding the parental influence on a child’s PA motivation and behaviors regarding young populations with disabilities, especially ASD children and adolescents [28]. Current evidence, though limited, suggests that when parents actively encourage, support, and facilitate PA, children and youth with disabilities tend to be more physically active [28,29,30,31]. Emerging evidence also indicates that parental encouragement of PA functions as a broader relational resource for families of children with disabilities. For example, pandemic-era surveys show that stronger parental PA encouragement is associated with healthier daily routines and less screen-time among children and youth with diverse disabilities, including large ASD subsamples [29,31]. Complementary qualitative accounts from parents of adolescents on the spectrum suggest that praise, collaborative planning, and shared activity not only promote exercise but also may alleviate behavioral challenges and enhance overall family well-being [32].

However, findings are not uniform, as at least one early seminal study on PA determinants in ASD youth found no parental effects whatsoever [33]. Compared with the rich literature on typically developing children, research dealing with the parental influence on PA in disability populations, especially ASD youth, remains sparse. Additional research is warranted to elucidate the relationship between parenting variables and children’s PA, with particular emphasis on the mechanisms underlying these associations in disability-specific populations, such as individuals with Autism ASD. This line of inquiry is especially critical given that such populations are particularly vulnerable to sedentary lifestyles and associated psychological difficulties, which, in turn, place them at elevated risk for significant health impairments.

## 2. The Current Study

In light of notable gaps in the existing literature concerning the role of parenting in emotional and health-related outcomes among individuals on the autism spectrum, the present study seeks to address this void by examining the associations between parenting styles and practices, anxiety symptoms, and PA levels in a wide school-age range of individuals with ASD functioning at a moderate-to-high level. Given the potential linkage between PA and emotional functioning, particularly in relation to anxiety, the study aims not only to explore the possible parental influences on offspring with ASD, but also to investigate the direct association between PA levels and anxiety symptoms in this population. Employing a path analysis model, the study will test a set of hypotheses concerning the relationships among parenting styles (i.e., authoritative, authoritarian, and permissive), parental encouragement of PA (a key parenting practice highlighted in the literature and assumed to be especially relevant for this population), and the offspring’s levels of PA and anxiety. Furthermore, the model will examine potential mediating mechanisms that may shed light on how parenting styles and practices are related to both anxiety and PA in ASD youth, suggesting that authoritative and non-authoritative parenting would be inversely/indirectly related to these child’s outcomes via encouragement of PA. The latter constitutes an effective parental measure for enhancing offspring’s PA, which, especially in populations with disabilities, may also reflect general active and positive parent–child communication processes [29,30,31]. Hence, it is expected not only to be associated exclusively with higher PA but also with greater emotional well-being.

Below are the specific research hypotheses tested in the study using an integrative path model, which embody both the direct and indirect associations among the study’s variables:

**Hypothesis** **1.** *Parenting style would positively predict parental encouragement of PA, such that authoritative parenting would be uniquely associated with higher levels of encouragement, and non-authoritative styles (i.e., permissive and authoritarian) with lower levels.*

**Hypothesis** **2.** *Parenting styles would predict child anxiety both directly and indirectly via parental encouragement of PA, such that authoritative parenting would be uniquely associated with lower levels of anxiety and the non-authoritative styles (i.e., permissive and authoritarian) would be positively associated with higher levels.*

**Hypothesis** **3.** *Parenting styles would be indirectly associated with PA level among offspring with ASD, such that the differential indirect associations between the parenting styles (i.e., authoritative and non-authoritative) and the child PA score would be mediated by parental encouragement of PA.*

**Hypothesis** **4.** *Child PA score would be negatively and directly associated with the anxiety level among individual offspring with ASD.*

## 3. Method

### 3.1. Participants

The sample consisted of 76 parents of children diagnosed with ASD, including 54 parents of boys and 22 parents of girls. Participants were recruited as part of a broader study examining family and child characteristics in ASD. The children ranged in age from 6 to 18 years (*M* = 10.75, SD = 3.67), with no significant differences in age between boys and girls. ASD severity levels were comparable across groups (according to parents’ reports), with 54% of boys and 64% of girls classified as Level 1 (requiring support/high-functioning), and the remainder distributed across Level 2 (requiring substantial support/moderate-functioning) or unknown classifications; chi-square tests indicated no significant differences in ASD severity distribution by gender. The parents’ ages ranged from 23 to 65 years (*M* = 42.96, SD = 7.01), with no significant differences in age between groups. The majority of participating parents were mothers (76% overall), with a similar gender distribution across the two groups. Regarding family characteristics, there were no significant differences between groups in marital status, number of children in the household, or number of children with special needs. To examine the association between the child’s sex and ASD severity, we conducted a Fisher’s Exact Test. The result was not statistically significant, *p* = 0.789, indicating no significant association between sex and ASD severity. Similarly, the chi-square test yielded a non-significant result, *χ*^2^(1) = 0.154, *p* = 0.695. Table 1 describes in detail the sample’s demographics by child’s gender.

Taken together, analyses indicated no significant differences between demographic characteristics of autistic boys and girls in the current sample. Given the relatively small sample size, the demographic similarity between groups supports the pooling of data across child gender for subsequent analyses. This approach enhances statistical power and allows for more robust examination of the full sample without distinguishing by child gender.

### 3.2. Measures

**Parenting Styles and Dimensions Questionnaire (PSDQ).** The present study employed the short form of the Parenting Styles and Dimensions Questionnaire (PSDQ) [34] to assess participants’ parenting styles. This 32-item instrument was developed using Structural Equation Modeling based on data from 1900 mothers and fathers of preschool- and school-aged children [34]. The PSDQ has been widely utilized in research globally, particularly in studies involving parents of children in primary and middle school [35]. The questionnaire evaluates parents’ use of various practices, enabling classification into one of Baumrind’s [36] three parenting styles: authoritative (comprising subscales assessing warmth/support, regulation, and autonomy granting), authoritarian (comprising subscales of physical coercion, verbal hostility, and nonreasoning/punitive practices), and permissive (consisting of five items assessing indulgence). Parents respond using a 5-point Likert scale (1 = never, 5 = always), producing continuous scores for each parenting dimension, with higher scores reflecting greater use of practices characteristic of that style. The Hebrew version of the PSDQ employed in this study was translated, adapted, and validated by Yaffe [37] against other established measures of parenting styles. The internal consistency reliability coefficients obtained in the present study for the permissive (α = 0.70), authoritarian (α = 0.87), and authoritative (α = 0.88) parenting style scales are consistent with prior findings reported for both the original English version and the Hebrew adaptation [35,37,38].

**The Screen for Child Anxiety-Related Emotional Disorders (SCARED)** [39] is a 41-item instrument developed to assess anxiety symptoms in children, aligned with DSM-IV diagnostic categories. The parent report version of the SCARED was used in the current study to assess the anxiety level of the sample’s offspring with ASD. The instrument comprises five subscales measuring Panic/Somatic symptoms, Generalized Anxiety, Separation Anxiety, Social Anxiety, and School Avoidance. Items are rated on a 3-point scale (0 = not true, 2 = very true). Scores are summed to yield subscale and total anxiety scores. The SCARED has demonstrated robust psychometric properties and is widely used internationally. In this study, the SCARED overall scale exhibited excellent internal consistency reliability, as indicated by a Cronbach’s alpha coefficient of 0.95.

**Parental encouragement of physical activity PA** in ASD children was assessed using the *Encouragement from Parents Scale* [40,41] (7 items: e.g., “I encourage my child to exercise or be physically active”), based on the parent’s self-reports. The scale is used in the research literature for screening parental PA-related practices and involvement in the family environment. This parental practice’s score is accepted from averaging the seven related items, with a higher score representing greater parental PA influence in parent’s perception. The scale’s items were translated and adapted into Hebrew in previous work [25], which reported acceptable reliability indices for the Hebrew version. Consistently, the Cronbach’s alpha coefficient recorded in the current study was 0.85.

**Leisure-Time Physical Activity Questionnaire** [42]. The child’s PA was assessed using the Godin–Shephard Leisure-Time Physical Activity Questionnaire, a brief and widely used self-report instrument designed to estimate weekly engagement in PA of varying intensities. The questionnaire includes three categories of activity: strenuous, moderate, and mild, each assigned a corresponding metabolic equivalent (MET) value of 9, 5, and 3, respectively. A total weekly activity score is computed by multiplying the frequency of each activity category by its MET value and summing the products. For estimating health benefits, a health contribution score can also be calculated using only moderate and strenuous activities, in line with public health guidelines. The questionnaire has demonstrated acceptable validity and is considered particularly useful due to its simplicity and ease of administration, which makes it particularly suitable for assessing PA in populations with disabilities. Accordingly, in the current study we used parent’s reports on the child’s PA level in leisure time contexts.

### 3.3. Procedure

Data collection was conducted during the year 2024. Participants were recruited using a multi-stage strategy targeting parents of children diagnosed with ASD. Initial outreach was conducted personally by members of the research team and trained research assistants. Subsequently, a snowball sampling method was employed, in which participating parents were encouraged to distribute the study invitation among other families raising ASD children. Inclusion criteria required participants to be parents of at least one school-aged child with a formal diagnosis of ASD. Parents of children undergoing diagnostic evaluation without a confirmed ASD diagnosis, parents of preschool children, and parents of children with other primary diagnoses were excluded from the study.

Participants, parents of children and adolescents diagnosed with ASD, were invited to take part in the present study. The recruitment notice provided a detailed description of the study variables, background, and significance, as well as information regarding the nature of participation (i.e., completion of online questionnaires), the estimated time required, and the accompanying compensation. The invitation was initially distributed through the research team and subsequently shared among potential participants via family referral. The invitation included a comprehensive explanation of the study’s aims, which focused on examining relationships between parenting characteristics, parenting behaviors, PA, and emotional and behavioral aspects in offspring with ASD.

Participation involved completing a battery of questionnaires using a secure Google Forms platform. An informed consent form was embedded at the beginning of the survey. Only those who provided digital consent were able to proceed. Questionnaires were administered anonymously and in Hebrew. Upon completion of the survey, participants received compensation in the form of a gift voucher equivalent to approximately USD 10. Parents were asked to indicate how many children they had, how many of those children had a formal ASD diagnosis, and were instructed to complete the questionnaire set with reference to one school-aged ASD child. The level of functioning referring to their child was reported by parents based on their knowledge of the child’s abilities, which reflects the information from formal diagnoses and/or their day-to-day experiences. Participation was voluntary, and respondents were informed that they could withdraw from the study at any stage, either during or after questionnaire completion, particularly if they experienced emotional discomfort. The study received ethical approval from the Institutional Review Board (IRB) of the authors’ institution (Ref. No. 9-8/2023).

Before launching the full survey, a pilot study was conducted with 10 participants to assess the clarity of questionnaire items and instructions, the time required to complete the survey, and potential emotional burden. Participants in the pilot phase were asked to provide feedback on these aspects upon completing the survey. Feedback was used to refine the survey materials prior to full-scale distribution. Following data collection, raw data were downloaded and stored on password-protected computers belonging to the research team. Prior to statistical analysis, data underwent screening to ensure accuracy and completeness. This included checks for missing values, outliers, duplicate responses, and response consistency.

### 3.4. Statistical Analyses

Data were analyzed using SPSS v28 and AMOS v28 software. Descriptive statistics and zero-order correlations were computed to examine associations among study variables. Independent-sample *t*-tests were conducted to assess sex differences in parenting styles, parental encouragement of PA, and child outcomes. Path analysis was then performed to test the hypothesized direct and indirect relationships between parenting styles, parental encouragement of PA, and children’s PA and anxiety levels. The analysis used the combined sample (N = 76), as subgroup sizes, particularly for girls, were too small for separate modeling. Model fit was evaluated using standard indices (χ^2^, CFI, RMSEA, GFI, SRMR). Demographic variables, including parent and child age, number of children, and ASD severity, were examined as potential covariates but were not included in the final model due to nonsignificant associations with the study variables.

## 4. Results

### 4.1. Correlations and Mean Comparisons

Mothers’ and fathers’ reports were treated as a sample’s whole due to the relatively small number of father participants, which did not allow for separate analysis by parent gender. Table 2 indicates that parenting styles and practices of PA encouragement were correlated, with the authoritative parenting positively and moderately correlated with the latter variable and the non-authoritative parenting (i.e., authoritarian and permissive) negatively correlated with it. These correlational patterns were essentially consistent across child sex, although insignificant for boys with respect the non-authoritative styles. Non-authoritative, but not authoritative, parenting was positively correlated with child anxiety levels. Expectedly, parental encouragement of PA was positively and negatively correlated with their child’s PA score and anxiety level (respectively). These patterns were evident across both boys and girls, albeit in some correlational cases only approaching significance in the latter group, due to its small size. Finally, we recorded contrasting correlational directions between PA score and anxiety level between boys (*r* = 0.16, *p* = 0.27) and girls (*r* = −0.30, *p* = 0.17). Although the correlation pattern for girls is consistent with the prior speculation regarding these variables, it is still statistically insignificant due to this group’s small sample size.

As for the child’s sex differences in the research variables, Table 3 indicates that boys and girls are basically exposed to similar patterns of parenting styles, but significantly vary on their parents’ encouragement of PA. Consistently, the sample’s parents reported that ASD boys are significantly more physically active than girls, while seemingly tending to be less anxious (result only approaching significance possibly due to very small sample size, but with medium effect size).

**Figure 1:**
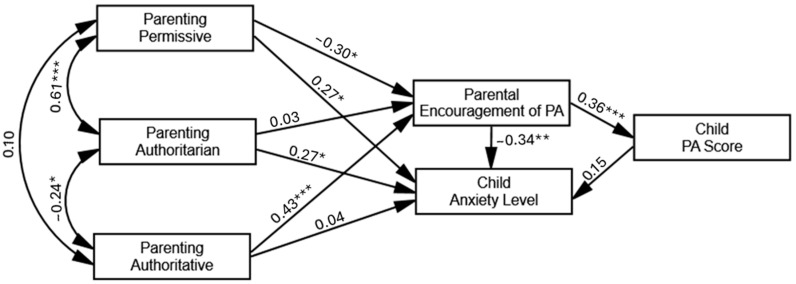
Path model describing the direct and indirect effects of parenting styles and parental PA encouragement on the child’s PA score and anxiety level (indices represent standardized estimates). * *p* ≤ 0.05, ** *p* ≤ 0.005, *** *p* ≤ 0.001.

The research hypotheses are embodied in an integrative path model (Figure 1) describing the direct and indirect associations between the parent and the child research variables. Since the sample comprises small subgroup sizes, especially within the girls’ group (*n* = 22), performing separate path analyses by child’s sex was statistically impractical due to insufficient statistical power and potential instability of the model estimates. Therefore, we conducted the path analysis using the combined sample (*N* = 76) without differentiating by sex, after ensuring general equivalence in structural weights across child sex (CMIN-*χ^2^*(9) = 10.33, *p* = 0.85). This approach is justified in the context of the current sample, given the inherent sex prevalence differences and the demographic similarity between these groups. The path model yielded good model fit properties (*χ^2^*(3) = 0.90, *p* = 0.83; comparative fit index [CFI] = 0.999, root-mean-square error of approximation [RMSEA] = 0.001, goodness-of-fit index [GFI] = 0.996, standardized root-mean-square residual [SRMR] = 0.022), although it may be saturated or nearly saturated and thus expected to yield very good fit indices by design.

The following demographics were considered as possible control variables, including the parent’s and child’s ages, the number of children in the family, and the number of siblings with ASD in the family. We found no significant statistical relationships between these demographics and the study variables (apart from the child’s age, which we considered in the study variables in Table 2; these non-significant correlations are deliberately not detailed here), which eliminated the need to control their effects on the model’s dependent variables. We also examined the potential effect of the child’s ASD severity on the dependent variables, as it was reported by parents based on a two-level functional classification. Although differences in PA scores (*M_difference* = 7.47, *p* = 0.18) and anxiety levels (*M_difference* = 6.12, *p* = 0.16) between the medium-(i.e., level 2) and high-functioning (i.e., level 1) ASD groups appeared in the expected direction, they were not statistically significant. Furthermore, including ASD severity as a control variable in the path model had a negligible impact on both the path coefficients and the model fit indices. To maximize the sample size and retain cases with missing data on ASD severity, we present the original model without including this variable as a covariate.

### 4.2. Hypothesis Testing

Hypothesis 1 stated that parenting style would predict parental encouragement of PA, such that authoritative parenting would be uniquely associated with higher levels of encouragement, and non-authoritative styles with lower levels. This hypothesis was partially supported by the data (Figure 1), with authoritative parenting significantly and positively associated with encouragement of PA and permissive parenting negatively associated with it. Authoritarian parenting, however, was not uniquely associated with parental encouragement of PA.

Hypothesis 2 stated that parenting styles would predict child anxiety both directly and indirectly via parental encouragement of PA, such that authoritative parenting would be negatively associated with the anxiety level and the non-authoritative styles would be positively associated with it. The present hypothesis was partially confirmed both at the direct and indirect levels. At the direct level, we found positive associations between non-authoritative parenting and child anxiety levels, in which permissive and authoritarian styles were related to higher anxiety levels in ASD children. Authoritative parenting was not directly/uniquely associated with child anxiety levels. At the indirect level, however, authoritative parenting was significantly associated with child anxiety, with parental encouragement of PA as mediator (*β* = −0.12, *p* = 0.008), possibly suggesting that autistic children of authoritative parents are less anxious partially due to their parents’ being more encouraging regarding PA. Conversely, the indirect positive association between permissive parenting and child anxiety via parental encouragement of PA was also significant (*β* = 0.08, *p* = 0.02), while no significant indirect effect was recorded for authoritarian parenting on child anxiety.

Hypothesis 3 stated that parenting styles would be indirectly associated with PA level among offspring with ASD, such that the differential indirect associations between the parenting styles (i.e., authoritative and non-authoritative) and the child’s PA score would be mediated by parental encouragement of PA. The present hypothesis was confirmed for authoritative parenting, with its positive effect on the child’s PA score significantly and fully mediated by parental encouragement of PA (*β* = −0.16, *p* = 0.001). Namely, ASD children of authoritative parents are more physically active, possibly due to their parental encouragement of PA. Conversely, the negative effect of permissive parenting on child’s PA score was also fully mediated by parental encouragement of PA (*β* = −0.11, *p* = 0.02), meaning that ASD children of permissive parents are less physically active, possibly due to the less parental encouragement of PA. These association patterns were not significantly evident for authoritarian parenting.

Hypothesis 4 stated that PA score would be negatively and directly associated with the level of anxiety among individual offspring with ASD. This hypothesis was not supported by the results given the insignificant path coefficient found between PA score and anxiety, seemingly due to contrasting correlational directions across child’s sex (which were both insignificant).

## 5. Discussion

The study explores the relationships between parenting styles and practices and emotional and health outcomes in school-aged ASD children and adolescents. Specifically, the study focused on how authoritative and non-authoritative parenting and parental encouragement of PA explain physical activity scores and anxiety level in moderate-to-high functioning young individuals with ASD. This integrates two established bodies of research in the fields of child developmental and clinical psychology and health studies, including one that identifies parenting as a key etiological factor in the development of childhood anxiety [18], and another that demonstrates the central role of parental influences in shaping children’s physical activity [27]. Despite robust evidence in typically developing populations, empirical research on these relationships in disability groups, particularly among ASD youth, remains limited [28].

The study contributes two key findings that strengthen the existing bodies of literature: first, by establishing a link between parenting styles and both anxiety and PA levels in autistic children; and second, by partially explaining the specific mechanisms underlying these associations.

Specifically, a robust body of research has demonstrated that patterns of excessive parental control, including denial of autonomy and overprotection, as well as a lack of warmth, are associated with increased anxiety, particularly in typically developing children and adolescents [15,16,17,18]. We consistently found unique association between authoritarian parenting and elevated anxiety in young individuals with ASD. Despite limited previous evidence linking parenting to increased anxiety in ASD children [21,23], the current findings are among the first to demonstrate a specific association between an authoritarian parenting style, characterized by psychological control, strictness, and low warmth, and elevated anxiety in autistic children. ASD children and adolescents exhibit heightened vulnerability to anxiety due to a combination of social-communication impairments, heightened self-awareness, and difficulties in emotional regulation. These individuals may experience anxiety as a secondary effect of their core ASD symptoms, particularly in socially demanding situations, where their awareness of social deficits amplifies distress [43]. The overlapping features between ASD and anxiety, such as rigidity, avoidance, and obsessive behaviors, may bidirectionally contribute to intensifying functional impairment and anxiety levels [21,43]. Overcontrolling parenting, marked by excessive regulation of a child’s experiences and even overprotectiveness, may be particularly harmful in the context of anxiety, as it can deprive children of opportunities to develop autonomy and a sense of control [18,44,45]. This may be especially detrimental in these psychological and developmental contexts associated with children on the autism spectrum, whose inherent difficulties make them particularly vulnerable to anxiety-enhancing experiences, especially those involving early uncontrollable situations within parent–child interactions.

Furthermore, emerging research suggests that permissive parenting might play a significant role in shaping anxious patterns especially among young children and in youth with disabilities [22,46]. This was further corroborated by the current study’s findings on individual with ASD, showing unique association between permissive parenting and child’s elevated anxiety. Indeed, parenting characterized by minimal behavioral control and structure may lead to uncertainty and ambiguity regarding behavioral boundaries, causing children to feel insecure about expectations and consequences, thereby exacerbating anxious feelings [46]. Moreover, in families of ASD children, maladaptive parenting often results from heightened parental stress [47]; parents experiencing higher distress may adopt permissive strategies, such as avoiding discipline, to minimize immediate emotional strain. This lack of structure, however, limits the child’s opportunity to develop effective emotional regulation and coping skills, further intensifying emotional vulnerabilities and anxiety symptoms [22]. Thus, permissive parenting indirectly fosters a developmental environment where anxious behaviors may thrive, particularly in children already dealing with emotional regulation challenges due to disabilities.

While authoritative parenting was not directly associated with child anxiety, it was substantially associated with encouragement of PA in the sample’s parents, which, in turn, was associated uniquely with both lower child anxiety levels and a higher score of PA. This suggests that ASD children and adolescents of authoritative parents are less anxious and more physically active possibly due to their parents’ affinity to encourage them to be more physically active. As expected, the contrary case was evident for the permissive parenting (but not for authoritarian), as it was found to be uniquely associated with lower and higher levels of the child’s PA and anxiety (respectively) via lower PA encouragement. Thus, the parental practice of encouraging PA in families raising ASD children may constitute a possible mechanism explaining the relationship between parenting styles and child’s outcomes.

Even though no unique statistical association was observed between PA and anxiety in the current sample, practitioners might consider physical activity not only as a health-promoting behavior but also as a platform for strengthening parent–child relationships and reducing anxiety symptoms. While prior research indicates that PA interventions can enhance physical, social, and emotional functioning among ASD youth [6], these effects often emerge under structured and supportive intervention conditions. In naturalistic or unstructured daily contexts, variations in activity type, intensity, and social environment may limit the consistency of the association between PA and emotional outcomes (the present study’s reliance on general PA levels rather than intervention-based measures may therefore explain the absence of a significant association). Therefore, encouraging shared, structured physical activity routines may serve as a meaningful way to foster emotional regulation and communication within ASD families. While this mediating role is well-acknowledged in the research literature on parental influence on children’s physical activity in both typical and atypical developmental populations [26,27,28,29,30], its seemingly specific protective role in relation to child anxiety is intriguing and may be unique to this particular population. Consistent with our primary assumption, this finding may indicate that parental encouragement and support of physical activity functions as a broader relational and communicative resource for families of ASD children, one that may also influence emotional domains such as anxiety. In line with previous evidence suggesting that parental practices related to physical activity can affect broader aspects of functioning beyond physical activity itself [29,31,32], our findings may imply that, in the context of this specific population, parental encouragement of physical activity reflects a more profound, versatile, and expansive familial communicative dynamic, one that may even contribute to the child’s emotional well-being. To examine this notion further, more research is needed using broader methodological and conceptual approaches. Yet, the findings highlight the dual importance of parenting style and parental encouragement in shaping both physical activity and anxiety outcomes in ASD youth. Intervention programs targeting these families should integrate training that promotes authoritative parenting practices, marked by warmth, structure, and autonomy support, while also equipping parents with practical strategies to actively encourage physical activity. This combined approach may not only enhance children’s physical engagement but also may foster emotional regulation and reduce anxiety symptoms.

### Limitations and Directions for Future Research

While the present study offers novel insights into the relationships between parenting styles, PA encouragement, and emotional outcomes in ASD children and adolescents, several limitations should be acknowledged, alongside recommendations for future inquiry. A primary limitation of the study is its relatively small sample size (*N* = 76), which was constrained by the considerable difficulty in accessing and recruiting families of ASD children. Although the sample size enabled the application of advanced statistical modeling, it restricts the generalizability of the findings and reduces the statistical power of subgroup analyses. Future studies should aim to replicate and extend these findings with larger, more diverse samples that would allow more robust inferential statistics and subgroup comparisons. Due to the limited number of participating fathers, data from mothers and fathers were aggregated, precluding the examination of potential differences in parenting styles and practices across parent gender. Given emerging evidence suggesting differential parental roles and behaviors in families of ASD children [47,48], future research would benefit from oversampling fathers and enabling comparative analyses of maternal versus paternal influences. Exploring dyadic parent–child relationships using actor–partner interdependence models may further enhance our understanding of these dynamics. Another key limitation in this regard is the absence of dyadic parental data. Parenting style and parental PA encouragement were reported by only one parent per family, preventing examination of inter-parent agreement or discrepancy and their effects on child outcomes. Recruiting both caregivers of autistic youth is challenging due to caregiving demands, lower participation among non-primary caregivers (often fathers), and single-caregiver or separated households. As a result, potential dyadic influences and shared-method bias cannot be ruled out, and parent-within-family comparisons were not possible. Future research should include both caregivers and use dyadic and multi-informant designs to better capture family dynamics.

As for child gender, although the study explored potential sex differences among children, the small sample size in the female subgroup (*n* = 22) did not permit independent path analyses by child gender. While we ensured measurement equivalence across child sex and confirmed that structural weights did not differ significantly, collapsing data across genders may have masked meaningful interaction effects. Importantly, despite this limitation reflecting broader epidemiological trends in ASD prevalence, the observed group differences in PA and anxiety suggest that gender-specific pathways warrant more nuanced investigation. Future studies should strive for balanced gender representation and consider employing stratified sampling or targeted recruitment strategies to allow meaningful multi-group modeling.

A further methodological limitation lies in the study’s cross-sectional design, which precludes definitive conclusions regarding the directionality or causality of the observed associations. While the path model tested theoretically grounded mediational relationships, such as the indirect effects of parenting styles on child outcomes via parental encouragement of PA, such models imply, but do not confirm, causal processes. Mediation analyses conducted on cross-sectional data are inherently limited, as they assume temporal ordering and underlying change processes that are not empirically observed [49]. Consequently, although the findings are consistent with theoretical frameworks suggesting causal pathways, alternative explanations such as reverse or bidirectional effects cannot be ruled out. Longitudinal or experimental research designs are essential to verify these proposed mechanisms and to clarify the developmental sequencing of parenting, PA encouragement, and emotional outcomes in ASD children.

Finally, another methodological limitation concerns the reliance on a single informant parent as the sole source of data for both independent and dependent variables. This approach was necessitated by the challenges of collecting self-report or observational data from ASD children, particularly those young children with limited verbal and cognitive skills. However, shared-method variance may artificially inflate associations between variables [50], raising concerns about the validity of the results. To address this limitation, future research should incorporate multi-informant designs, including teacher reports, clinician assessments, and, where feasible, child self-reports or behavioral observations.

In conclusion, while this study contributes valuable empirical evidence to a relatively underexplored domain, its findings should be interpreted in light of these methodological constraints. Addressing these limitations in future research will be essential to developing more comprehensive, culturally sensitive, and practically relevant models of parenting and child adjustment in the context of ASD.

## Figures and Tables

**Table 1:** Sample description by child gender.

Variables	Subscale	ASD Boys (*n* = 54)	ASD Girls (*n* = 22)	Statistics Chi-Square/t	*p*
Parent gender	Males	13 (24%)	5 (23%)	*χ*^2^(1) = 0.016	0.90
	Females	41 (76%)	17 (77%)		
Parent age	Range	32–65 (*M* = 42 ± 6.34)	23–62 (*M* = 45 ± 8.1)	*t* = 1.64	0.11
Child age	Range	6–18 (*M* = 10.36 ± 3.65)	6–18 (*M* = 11.70 ± 3.62)	*t* = 1.64	0.15
ASD severity	Level 1 (high)	29 (54%)	14 (64%)	*χ*^2^(2) = 0.154	0.69
	Level 2 (med)	16 (30%)	6 (27%)		
	Unknown	9 (16%)	2 (9%)		
Family status	Married	39 (72%)	14 (63.6%)	*χ*^2^(2) = 1.52	0.68
	Divorced	6 (11%)	4 (18.2%)		
	Other	9 (17%)	4 (18.2%)		
Family number of children	Range	1–6 (*M* = 2.61 ± 1.14)	1–5 (*M* = 2.68 ± 1.04)	*t* = 0.25	0.80
Family number of children with special needs	Range	1–3 (*M* = 1.25 ± 0.55)	1–3 (*M* = 1.43 ± 0.68)	*t* = 1.21	0.23

**Table 2:** Descriptive statistics and zero-order correlations of the study’s continuous variables.

	1	2	3	4	5	6	7
*Parent* *variables*							
1. Permissive parenting	-	0.62 **	0.1	0.23 *	0.01	0.51 **	0.16
2. Authoritarian parenting		-	−0.24 *	−0.25 *	−0.05	0.49 **	−0.17
3. Authoritative parenting			-	0.40 **	0.12	−0.11	−0.05
4. Encouragement of PA				-	0.36 **	−0.39 **	0.01
*Child Variables*							
5. PA score					-	0.02	−0.15
6. Anxiety level						-	0.01
7. Child age							-
Mean	3.03	1.99	3.99	3.49	30.25	49.07	10.75
SD	0.78	0.71	0.64	0.88	22.06	18.15	3.67

* *p* ≤ 0.05, ** *p* ≤ 0.001.

**Table 3:** Means, standard deviations, and the t-values (powered by Cohen’s *d*) for the differences between boys and girls in the study variables.

	Boys (*n* = 54)		Girls (*n* = 22)				
Study Variables	*M*	*SD*	*M*	*SD*	*t*	*p*	Cohen’s *d*
*Parent* *variables*							
Permissive parenting	3.02	0.87	3.05	0.79	0.21	0.84	0.05
Authoritarian parenting	1.96	0.75	2.08	0.63	0.69	0.5	0.17
Authoritative parenting	3.99	0.64	3.98	0.64	0.07	0.94	0.02
Encouragement of PA	3.6	0.88	3.21	0.84	1.96	0.05	0.45
*Child variables*							
PA score	34.13	23.47	20.73	14.64	3.01	0.004	0.63
Anxiety level	46.94	18.64	54.27	16.11	1.61	0.11	0.41

*Introducing a path model testing the relationships between parenting styles and PA encouragement and autistic child’s PA and anxiety levels.*

## Data Availability

Available from the authors upon reasonable request due to ethical and privacy considerations that are necessary to protect the confidentiality of the participants.
